# Correction: Biting the bullet: When self-efficacy mediates the stressful effects of COVID-19 beliefs

**DOI:** 10.1371/journal.pone.0265330

**Published:** 2022-03-09

**Authors:** Natanya Meyer, Thomas Niemand, Andrés Davila, Sascha Kraus

## Notice of republication

This article was republished on February 25, 2022, to correct the order in which Dr. Kraus’ affiliations are listed. The publisher apologizes for the error. Please download this article again to view the correct version. The originally published, uncorrected article and the republished, corrected articles are provided here for reference.

## Supporting information

S1 File Originally published, uncorrected article. (PDF)

S2 File Republished, corrected article. (PDF)
