# Supplementary material for: Biting the bullet: When self-efficacy mediates the stressful effects of COVID-19 beliefs
Source: PLoS One. 2022 Jan 28;17(1):e0263022. doi: 10.1371/journal.pone.0263022 (PMC8797252; doi:10.1371/journal.pone.0263022)
Supplement: S1 Appendix — (DOCX) [file pone.0263022.s001.docx]

**S1 Appendix: Scale Items**

| **Item No.** | **Item Statement** | **Adapted from** |
| --- | --- | --- |
| **Information seeking (IV)** | | |
| 1 | I am interested in looking up information about COVID-19. | Prigge et al. [54]** |
| 2 | I regularly look for information about COVID-19. |  |
| 3 | I frequently look for information about COVID-19. |  |
| 4 | I spend a lot of time collecting information about COVID-19. |  |
| 5 | Compared to others, I would call myself an expert on COVID-19. |  |
| 6 | I am as well informed as most health professionals about current COVID-19 knowledge. |  |
| 7 | I regularly analyze any new information about COVID-19. |  |
| 8* | I would be able to make the right decision if I had the first symptoms of COVID-19 (fever, shortness of breath). |  |
| 9* | If I had symptoms, I would be able to evaluate them and take the most appropriate decision (call my doctor, stay home, ...). |  |
| 10* | I could take the necessary steps in case of COVID-19. |  |
| **Perceived invulnerability (IV)** | | |
| 1 | I am less likely than most people to get COVID-19. | Rhodes & Arceo [65] |
| 2 | There is little chance that I could get or spread COVID-19 from what I do in my everyday life. |  |
| 3 | I am not at risk for getting infected with COVID-19. |  |
| 4 | People like me don't get COVID-19. |  |
| 5 | My body could fight off COVID-19 infection. |  |
| **Perceived disruption (IV)** | | |
| 1 | Having COVID-19 would be disruptive to my social life. | Gerend, Shepherd & Monday [66] |
| 2 | Having COVID-19 would be disruptive to my physical health. |  |
| 3 | Having COVID-19 would be disruptive to my everyday life. |  |
| 4 | Having COVID-19 would be disruptive to my life overall. |  |
| **Health importance (IV)** | | |
| 1 | My health is my top priority. | Prigge et al. [54] |
| 2 | I am concerned about my health and am taking action to prevent COVID-19. |  |
| 3 | Taking care of my health means a lot to me. |  |
| **Response effectiveness (IV)** | | |
| 1 | Washing your hands frequently is an effective method for avoiding COVID-19. | Van Empelen, Schaalma, Kok & Jansen [67] |
| 2 | Wearing a surgical mask is an effective method for avoiding COVID-19. |  |
| 3 | Staying at home is an effective method for avoiding COVID-19. |  |
| 4 | Practicing social distancing is an effective method for avoiding COVID-19. |  |
| 5 | Avoiding crowds is an effective method for fighting COVID-19. |  |
| **Self-Efficacy (M)** | | |
| 1 | When I am faced with a new situation, I know how to deal with it. | Prigge et al. [54] |
| 2 | I can usually think of a solution if I am in trouble. |  |
| 3 | Whatever comes my way, I can handle it. |  |
| **Perceived Stress during COVID-19 (DV)** | | |
| 1 | Since the COVID-19 epidemic began, I have been upset because of something that happened unexpectedly. | Cohen, Kamarck & Mermelstein [53] |
| 2 | Since the COVID-19 epidemic began, I have felt unable to control the important things in my life. |  |
| 3 | Since the COVID-19 epidemic began, I have felt nervous and stressed. |  |
| 4^I^ | Since the COVID-19 epidemic began, I have felt confident about my ability to handle my personal problems. |  |
| 5^I^ | Since the COVID-19 epidemic began, I have felt that things are going my way. |  |
| 6 | Since the COVID-19 epidemic began, I have found that I cannot cope with all the things that I have to do. |  |
| 7^I^ | Since the COVID-19 epidemic began, I have been able to control irritations in my life. |  |
| 8^I^ | Since the COVID-19 epidemic began, I have felt that I am on top of things. |  |
| 9 | Since the COVID-19 epidemic began, I have been angered because of things that happened that were outside of my control. |  |
| 10 | Since the COVID-19 epidemic began, I have felt difficulties are piling up so high that I cannot overcome them. |  |
| **Big 5 personality traits** | | |
| Big Five – Agreeability | | John & Srivastava [55] |
| 1^I^ | I can be cold and distant with people. |  |
| 2 | Helping others gives me a sense of satisfaction. |  |
| 3^I^ | I distrust most people. |  |
| 4^I^ | People tell me I have a negative attitude. |  |
| **Big Five – Conscientiousness** | |  |
| 1^I^ | I can be disorganized. |  |
| 2 | I usually work hard. |  |
| 3^I^ | I have trouble starting tasks. |  |
| 4 | I always know where to find my things. |  |
| **Big Five – Extraversion** | |  |
| 1^I^ | I tend to be shy in social situations. |  |
| 2 | I like going out and meeting people. |  |
| 3 | I enjoy meeting new people at social events. |  |
| 4^I^ | I have trouble making small talk with new people. |  |
| **Big Five – Openness** | |  |
| 1 | I think of myself as open-minded. |  |
| 2^I^ | I like routine work. |  |
| 3 | I am interested in other cultures and ways of life. |  |
| 4 | I need variety in my daily life. |  |
| 5 | I seek out new experiences. |  |
| 6^I^ | I like things to stay the same. |  |
| **Big Five – Stability** | |  |
| 1 | I am generally calm and relaxed. |  |
| 2^I^ | My moods tend to change a lot from one moment to another. |  |
| 3 | I stay calm in most circumstances. |  |
| 4 | I can easily control my emotions. |  |

Notes. * Item omitted due to low factor loading, **: Originally, the items were derived from information search and knowledge development sub-dimensions. This two-dimensional perspective was not confirmed. We hence use the term "Information seeking" resembling both dimensions in one. I: Inverted item
