# Supplementary material for: Correction: Biting the bullet: When self-efficacy mediates the stressful effects of COVID-19 beliefs
Source: PLoS One. 2022 Mar 9;17(3):e0265330. doi: 10.1371/journal.pone.0265330 (PMC8906615; doi:10.1371/journal.pone.0265330)
Supplement: S1 File — (PDF) [file pone.0265330.s001.pdf]

PLOS ONE

RESEARCH ARTICLE

Biting the bullet: When self-efficacy mediates the stressful effects of COVID-19 beliefs

Natanya Meyer[1], Thomas Niemand[2], Andrés Davila[3], Sascha Kraus[1,4]*

1 Department of Business Management, DHET-NRF SARChI Entrepreneurship Education, University of Johannesburg, Auckland Park, South Africa, **2** Department of Market Research, Institute of Management and Economics, Clausthal University of Technology, Clausthal-Zellerfeld, Germany, **3** ESCE International Business School, École Supérieure du Commerce Extérieur & Praditus SAS, Paris, France, **4** Faculty of Economics and Management, Free University of Bozen-Bolzano, Bolzano, Italy

* sascha.kraus@zfke.de

Check for updates

Abstract

The impact that COVID-19 had on individuals globally has been immense. Our study aims to determine if the various COVID-19 related beliefs (information seeking; invulnerability; disruption; health importance and response effectiveness) are predictors of perceived stress and if self-efficacy acts as a mediator in reducing perceived COVID-19 related stress. From a large sample of 23,629, data were assessed using validated multi-item measures for seven COVID-19 related beliefs, self-efficacy and perceived stress. After conducting a series of tests and checks via Confirmatory Factor Analyses, linear modelling and mediation analyses with bootstrapping were applied to test direct and mediation hypotheses. It is found that stress perception is most strongly affected by self-efficacy and perceived disruption. Except for information seeking, which positively affected perceived stress, self-efficacy partially mediates all other COVID-19 related beliefs (perceptions of disruption, health importance and response effectiveness) in conjunction with their direct effects. Only perceived invulnerability elicited opposite effects on stress, increasing stress directly but decreasing stress indirectly by increasing self-efficacy. This finding gives reason to believe that individuals may disclose that they are less vulnerable to COVID-19, fostering their self-efficacy, but still accept that stressing factors such as economic and social consequences apply. Overall, reinforcing self-efficacy was carved out as the most important resilience factor against perceiving high levels of stress. On this basis, implications for research and practice are provided.

OPEN ACCESS

Citation: Meyer N, Niemand T, Davila A, Kraus S (2022) Biting the bullet: When self-efficacy mediates the stressful effects of COVID-19 beliefs. PLoS ONE 17(1): e0263022. https://doi.org/10.1371/journal.pone.0263022

Editor: Andrea Fronzetti Colladon, Universita degli Studi di Perugia, ITALY

Received: September 2, 2021

Accepted: January 10, 2022

Published: January 28, 2022

Copyright: © 2022 Meyer et al. This is an open access article distributed under the terms of the Creative Commons Attribution License, which permits unrestricted use, distribution, and reproduction in any medium, provided the original author and source are credited.

Data Availability Statement: The data are available at https://dx.doi.org/10.17632/b8zxmw5z3g.1.

Funding: This work was supported by the Open Access Publishing Fund provided by the Free University of Bozen-Bolzano. The funders had no role in study design, data collection and analysis, decision to publish, or preparation of the manuscript.

Competing interests: The authors have declared that no competing interests exist.

Introduction

March 11th, 2020 marked the date that changed the lives of millions of individuals globally [1]. Since the World Health Organization (WHO) announced the novel coronavirus SARS-CoV-2 causing the related disease, hereafter COVID-19, as a *pandemic*, the aftermath is still being experienced. As of August 2021, 199 million global cases and 4.2 million deaths have been reported. Countries such as the United States of America, Brazil, France, Spain, Netherlands, Czechia, Slovenia, and Sweden were some of the worst affected when considering the total

number of cases per 1 million population [2]. The impact of the pandemic affected everyone in some way.

Research has shown that individuals' psychological responses during serious life events, such as a war or pandemic, play a critical role in shaping its outcome [3, 4]. In the case of a pandemic, both the spread of the disease and the manifestation of emotional distress and social disorder throughout and after the event directly affects the population. Despite this, adequate solutions to manage or diminish pandemics' impact on mental health and well-being are still under-researched and not considered as important as physical health [5]. Taylor [6] opines that many healthcare resources are made available during pandemics, including the development and distribution of vaccines and testing and information centres. However, most healthcare agencies neglect or underestimate the importance of allocating resources devoted to pandemic-related emotional aspects (e.g., anxiety, fear, stress). While one would understand that aspects such as reducing transmission and further outbreaks are critical in the early stages of such pandemics, psychological and mental health requirements should not be neglected. The importance of understanding phycological factors is twofold. Firstly, it provides insights into how individuals deal with aspects relating to stress, fear and anxiety during serious life events. Second, it assists in understanding and managing bigger societal problems associated with stressful events such as stigmatization and xenophobia [6].

Despite continuous efforts to stop the spread of COVID-19, the emergence of new variants results in increased infection rates, causing uncertainty across nations. With increased fear of contracting the virus, possible long-term health concerns, job losses, and insecurity about future economic stability, individuals' heightened sense of insecurity has been observed. This uncertainty creates a perfect storm for increased stress and anxiety levels. Based on previous research on COVID-19 related beliefs [7] and self-efficacy theory [8], we argue that self-efficacy plays a key mediating role in stress perception and coping with stress. For example, perceiving severe disruption through COVID-19's consequences does not only cause stress per se, but it may also deteriorate an individual's belief to cope with the consequences–self-efficacy–causing even more stress and anxiety. Most studies on COVID-19 psychological factors (e.g., stress, fear, anxiety) were conducted on healthcare or frontline workers [9–11]. In addition, studies including individuals in a business context were limited. Torrès et al. [12] investigated health perceptions regarding French business owners' physical and mental health collected before and during the pandemic. Lathabhaven [13] considered economics, financial and depression anxiety as factors in a study on small business owners in India. To the best of our knowledge, the relationship between self-efficacy as a mediating role in stress perception and coping with stress has not been investigated regarding COVID-19. Still, it has substantial effects on employees, managers, founders, and other individuals in a business context. For example, these individuals may feel less able to apply for aid funds, rebuild businesses or approach new ventures. Using a large and broad sample capturing multiple countries, occupations and motives, we hence focus on the mediating effect of self-efficacy on perceived stress and propose the following research questions: *Do the various COVID-19 related beliefs (information seeking; perceived invulnerability; perceived disruption; health importance and response effectiveness) act as predictors of perceived stress*? *Does the level of self-efficacy act as a mediator in reducing perceived COVID-19 related stress*?

Theoretical framework and hypotheses development

COVID-19 related stress

Stress is an inevitable part of life. Authors such as Lazarus, Pearlin and Menaghan [14–16] conceptualized stress as a heightened state of emotions resulting from socio-environmental

demands that exceed a person's normal adaptive capacity. It further stems from the absenteeism of the required resources to achieve a certain outcome. Gmelch [17] defines stress as a demand on the body, both mentally and physically, which exceeds a person's capability to cope. Stress can be considered the outcome of exposure to a certain stimulus that can be considered threatening, harmful, or challenging, surpassing a person's ability to cope. Exposure to long-term stress may negatively affect a person's health and mental state [18]. The Centers for Disease Control and Prevention (CDC) state that the COVID-19 pandemic has been a major source of stress in individuals' lives [19]. Various authors opine that life events, such as those inflicted by COVID-19, act as stressors and are enhanced if individuals require changes in their ongoing life patterns [3, 11, 20]. Likewise, Aneshensel [21] referred to stressors as external situations or stimuli resulting in stress. When more than two stressors are active in a person's life at once, this may result in a strain that could have a more severe psychological impact on a person's well-being [22]. The COVID-19 pandemic has introduced several stressors into individuals' lives [10, 11, 13]. As mentioned, individuals are faced with a number of stress-related factors induced by the pandemic—ranging from health concerns to insecurity about future economic stability [6]. This leads to increased perceived stress and ultimately amplified stress levels.

Predictors of perceived COVID-19 related stress

In a large study of COVID-19 related beliefs with over 8,000 recipients, Clark et al. [7] found multiple factors that may predict voluntary compliance behaviors. We extend this study by adding stress as an outcome variable and self-efficacy as a mediator. For the reasons explained below, we focus on information seeking, perceived invulnerability, perceived disruption, health importance and response effectiveness as possible predictors of self-perceived COVID-19 related stress.

Information seeking and stress. Information seeking can be defined as the sourcing of information from selected outlets [23]. Case [24] mentions that information seeking is a behavior generally not acted upon until such time a situation forces one to do so. Although traditional theory on information seeking refers to it as having a positive effect on a person's state of mind, the amount of information available today, in some cases negative and conflicting, may have the opposite effect [25–27]. For example, Lambert and Loiselle [28] state that seeking information, specifically regarding health issues, may act as a coping strategy towards promoting better health. However, Yang and Kahlor [26] state that individuals may avoid information seeking in cases of negative conditions to reduce anxiety and stress. More specifically, Moreno et al. [27] refer to crisis and risk communication during pandemics which actively raises awareness of the magnitude and nature of associated risks. This type of information, in many instances, can create a heightened sense of anxiety or stress among certain individuals. As mainstream media reported COVID-19 situations almost constantly, avoidance to prevent anxiety was not always possible. Brasher [29] notes that information on negative aspects may cause more uncertainty resulting in fear, anxiety, and other adverse emotions. Yang and Kohler [26] opine that seeking information under extreme conditions, like during a pandemic, may be overwhelming and lead to increased stress. Hence, given our focus on COVID-19 related stress, it seems plausible to integrate information seeking as a potential factor inducing stress. Subsequently, we formulated our first hypothesis as:

H1: Higher information seeking is associated with higher perceived stress during COVID-19.

Perceived disruption and stress. Perceived disruption refers to the negative consequences individuals associate with an event or outcome, such as COVID-19. Disruption is

defined as the action of preventing a certain outcome or event from progressing to its normal state. The term disruptive refers to a cause of trouble prohibiting something from continuing as usual [30]. The onset of the COVID-19 pandemic resulted in massive disruption in the so-called "usual" way of doing things. Several studies have empirically proven that disruption is linked to increased stress. For example, Jones and Butler [31] found that disruption in the usual family roles, i.e., sailors or soldiers having to give up their role as a family caregiver, acts as a stressor. Another study by Picou et al. [32] found that disruption caused by a natural or technological disaster leads to increased stress. Elliott [33] notes that the disruption in education caused by lockdowns has negatively affected various cohorts of school-going children and caused increased levels of stress. Interesting, Carney et al. [34] found that for individuals over 50 years, COVID-19 related disruption had limited effects on stress; however, for younger individuals, higher perceived COVID-19 related disruption led to higher stress levels. We understand disruption as an important interference factor for well-being, and so far, we hypothesize that:

H2: Higher perceived disruption is associated with higher perceived stress during COVID-19.

Perceived invulnerability and stress. Being vulnerable is often conceptualized as being exposed and sensitive to stressors caused by a hazardous event or situation, limiting one's capacity to adapt. The characteristics of these stressors include their magnitude, duration, frequency and extent of the hazard [35]. On the contrary, invulnerability refers to the notion of having a feeling of control over a hazardous event or situation. Perceived invulnerability refers to an individual's belief that they will not experience negative consequences from a specific event or situation. Thus, a feeling of reduced risk appears when making decisions concerning certain behaviors [36] in our case COVID-19 health-related. In line with Clark et al. [7], we argue that the main consequence of (in)vulnerability for COVID-19 is the threat to someone's health or the likelihood of developing a health problem. A large body of literature demonstrates that understanding the COVID-19 symptoms, self-perceived risk of getting ill, and fear are noticeable predictors of adopting preventive behaviors [37, 38]. Other studies pointed out that COVID-19 strongly influenced how individuals changed their behavior, i.e., washing hands regularly and applying social distancing [38, 39]. Actively changing one's behavior in such a manner that makes you believe it could have a positive outcome reduces perceived stress [40]. We understand vulnerability and invulnerability as opposite poles of the same issue and proceed with the latter. Hence, invulnerability serves as a first resilience factor, and we hypothesize the following:

H3: Higher perceived invulnerability is associated with lower perceived stress during COVID-19.

Health importance and stress. Health importance refers to how easily and actively individuals believe they can take action to care for their health. In the case of COVID-19, the effects of health importance seem to be double-barreled. Initially, COVID-19 threatens the individual's health directly, causing individuals who have higher levels of health endorsing behavior to be more stressed [41]. Subsequently, more stress has a negative effect on a person's health itself [42]. On the contrary, Clark et al. [7] found that individuals who believe that taking care of their health acted as a predictor of voluntary COVID-19 compliance leading to a sense of being in control. As such, individuals with high health importance may be more up-to-date about the differential consequences of COVID-19 and tend to live a life that is less likely to result in severe symptoms, hospitalization or Long COVID, thereby reducing stress [43]. Balancing both positions, we assume that the latter contributes to coping with COVID-19 more effectively and exceeds the stress-inducing health effect. Thus, perceived health importance serves as a second resilience factor against stress, leading to the following hypothesis:

H4: Higher health importance is associated with lower perceived stress during COVID-19.

Response effectiveness and stress. Response effectiveness beliefs describe the perceived effectiveness of a recommended response to avoid a threat, in this case, coming down with COVID-19. Several studies revealed that most frontline workers were faced with increased stress due to additional protocols, inefficient instructions, being face-to-face with infected patients and a lack of personal protective equipment [11, 44, 45]. Oh et al. [44] also found that nurses experienced lower stress levels when the supply of protective wear and training was higher. Further studies showed that one of the most stressful factors among frontline workers was the lack of proper protocols [46]. All of these protocols have resulted in some reduction of COVID-19 transmission, even if only marginal. In a nutshell, it becomes evident that the belief of practicing these protocols or responses actually works against transmission of the virus and hence will reduce an individual's stress levels which acts as another resilience factor. This leads us to the next hypothesis:

H5: Higher response effectiveness is associated with lower perceived stress during COVID-19.

Self-efficacy and stress. Bandura [8] defined self-efficacy as a self-evaluation of one's competency to effectively perform a specific action needed to achieve a desired state or outcome. Zajacova et al. [47] add that self-efficacy works as a personality-specific trait and encapsulate it as a broad trust in one's capabilities and proficiencies to impact challenges in an effective manner, hence exhibiting a sense of coping. More self-efficacious individuals may face challenges better and thus feel less stressed [48]. Past research has unanimously confirmed that possessing high levels of self-efficacy decreases a person's possibility of experiencing stress [9, 10, 47, 49]. Self-efficacy increases one's sense of being in control of a situation one may encounter and can thus be seen as a stress-coping strategy [8]. As opposed to actually being in control, the perception of feeling in control may act as an important safeguard against negative stress. Based on these arguments, we pose the following hypothesis:

H6: Higher self-efficacy is associated with lower perceived stress during COVID-19.

We further argue that self-efficacy serves as a key variable in the process of appraisal and acts as a mediator between beliefs and stress (in our case, COVID-19 related). This can be argued based on the transactional theory of stress and coping to contextualize self-efficacy and stress, developed by Lazarus [14]. This theory states that the mediating role of appraisal influences the severity of stress or the reaction to it. Appraisal can also be understood as a cognitive process through which meaning is attributed to an outcome event (in our case, COVID-19). Simplified, the previous predictors are potential ways used to deal with or perceive COVID-19 related stressors (e.g., seeking information, disruption and invulnerability, perceiving it as important and effectively controlled) in a primary appraisal process. Self-efficacy then serves in a second appraisal process of the stressor having the competency to cope with the consequences of COVID-19. Consequently, self-efficacy becomes vital in assessing the challenges emanating from a certain stressful situation [50]. Beliefs shape not only the perception of the stressor but also how we evaluate our competency to cope with the stressor. Hence, it is plausible to assume that self-efficacy itself is influenced by the predictors [51], converting it into the role of a mediator (and not as an independent moderator). Please note that this implies partial mediation, as the direct effects (H1–H6) are still likely to exist. We consequently formulate the following hypotheses:

H7a: Self-efficacy mediates the relationship between information seeking and perceived stress during COVID-19.

H7b: Self-efficacy mediates the relationship between perceived disruption and perceived stress during COVID-19.

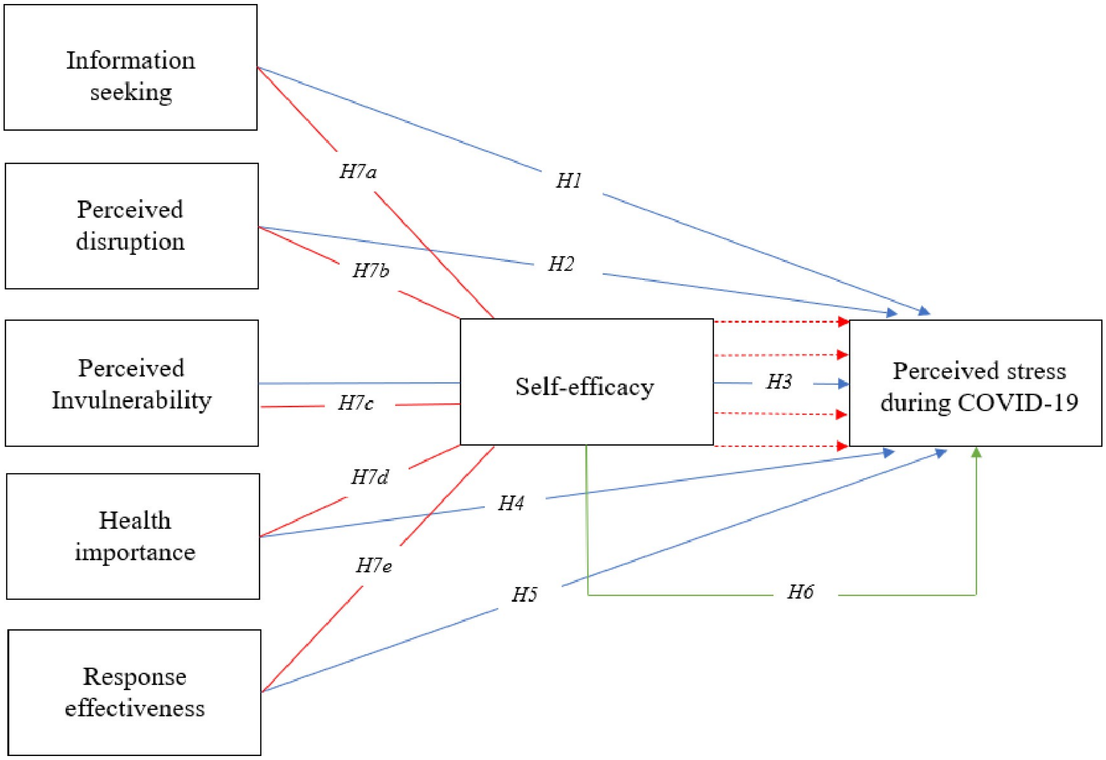

Fig 1. Theoretical framework.

https://doi.org/10.1371/journal.pone.0263022.g001

H7c: Self-efficacy mediates the relationship between perceived invulnerability and perceived stress during COVID-19.

H7d: Self-efficacy mediates the relationship between health importance and perceived stress during COVID-19.

H7e: Self-efficacy mediates the relationship between response effectiveness and perceived stress during COVID-19.

Based on the discussion above, we propose the following theoretical framework (Fig 1):

Methods

Sample and data collection

Our data was collected by the French-based talent-management platform Praditus [52]. Praditus allows individuals to obtain an assessment of their personality based on worldwide surveys capturing multiple topics and a variety of pre-validated measures. Hence, our data contains a subset of variables from the platform's surveys, and we only used the variables related to COVID-19 beliefs, stress, self-efficacy as well as the control variables gender, age, work experience and the Big 5 personality traits (agreeability, conscientiousness, extraversion, openness, and stability).

Measures and instruments

Table 1 depicts details of the variables included in our analysis. For predicting COVID-19 beliefs, we used the multi-item measures proposed and validated by Clark et al. [7]. Perceived stress was measured based on the 10 item-inventory [53], while for COVID-19 related self-

Table 1. Study variables.

Variable*	Variable type	Nr of items	Reliability[1]	Description
Information seeking	Independent/ Predictor	10	0.89 (0.40–0.89)	Information seeking can be defined as the intentional sourcing of information from selected information outlets (in this case, regarding COVID-19).
Perceived invulnerability	Independent/ Predictor	5	0.68 (0.41–0.69)	Perceived invulnerability reflects an individual's belief about the likelihood of a health threat's occurrence or the likelihood of developing a health problem (in this case, COVID-19).
Perceived disruption	Independent/ Predictor	4	0.85 (0.65–0.87)	Perceived disruption refers to the negative consequences an individual associates with an event or outcome, such as COVID-19. Thus, how much an event will disrupt a person's daily life. These consequences may relate to an anticipated event that may occur in the future or to a current state, such as a pre-existing health problem.
Health importance	Independent/ Predictor	3	0.74 (0.55–0.83)	Health importance refers to how easily and actively individuals believe they can take action to take care of their health.
Response effectiveness	Independent/ Predictor	5	0.80 (0.54–0.77)	Response effectiveness beliefs measure the perceived effectiveness of the recommended response to avoid the threat (COVID-19).
Self-efficacy	Mediator	3	0.75 (0.66–0.77)	Self-efficacy refers to individuals' estimation of their capability and effectiveness in performing a specific task well within the actual COVID-19 situation.
Perceived stress during COVID-19	Dependent/ Outcome	10	0.86 (0.45–0.78)	Perceived stress measures the extent to which individuals feel that their demands exceed their ability to cope.

*For further details regarding the variables, please refer to the Annexure. 1: Cronbach's Alpha, Lowest and highest factor loading from CFA in ().

https://doi.org/10.1371/journal.pone.0263022.t001

efficacy, we used a 3 item-scale [54]. As control variables, we included the primary Big 5 personality traits (agreeability, conscientiousness, extraversion, openness, and stability) based on the inventory of John and Srivastava [55] to safeguard against the issue that the effects are attributed to personal differences. All responses regarding these measures were captured on a five-point Likert-type scale ranging from 1 (completely disagree) to 5 (completely agree). Further, we used dummy variables for gender (women) and work experience (less than 1, 1 to 5, 6 to 10, more than 10 years) as well as standardized age as control variables, again to increase the robustness of our results.

Ethical statement

All data used in this study was collected ethically and with full consent by the participants. Participants were made aware that the data will be used for academic publications and ensured the protection thereof under the protocols provided by the General Data Protection Regulation (GDPR) (EU Regulation 2016/679) of the European Commission of 27 April 2016. No data of a sensitive matter was collected, all data was used in aggregate and anonymized, and no personal identifiers were reported. Praditus approved the data collection process. Full details of the privacy and data collection policy can be viewed at https://www.praditus.com/en/privacy-policy/.

Results

Demographics and descriptives

The sample comprised 23,629 participants, of which 67.1% (n = 15,866) were women, 30.3% (n = 7,172) men, 0.9% (n = 199) non-binary and 1.7% (n = 392) preferred not to disclose this information. Participants indicated to be primarily of French (28.7%; n = 6,776), US (11.2%; n = 2,568), German (10.6%; n = 2,504), Italian (6.5%; n = 1,528), Mexican (4.3%; n = 1,012)

and Spanish (4.2%; n = 990) nationality. The mean age of respondents was 28.50 years (SD = 10.60). All participants took part voluntarily and without financial compensation.

Validity checks

Before we confirm our theoretical framework and hypotheses, we conducted three additional tests to assess the reliability and validity of our multi-item measures. First, we performed Confirmatory Factor Analysis (CFA) followed by checking the impact of Common Method Bias and lastly exploring that participants do not differ as a function of non-response bias [56]. All analyses hereafter were conducted in R, most importantly using packages lavaan (CFAs) and mediation (mediations).

A CFA confirmed that the predictors, the outcome stress, and the mediator self-efficacy are measured reliably (Table 1). Only invulnerability scored slightly below the threshold of 0.7 (α = 0.68), which is identical to previous research [7]. We omitted three items from information seeking due to low loadings (which do not produce a reliable factor itself). The model fits the data reasonably well (Chi-squared = 56,499.24, df = 751, RMSEA = 0.06, SRMR = 0.06) [57]. All measures are discriminantly valid, applying the proposed procedure by Rönkkö and Cho [58], with the largest correlation among the measures being 0.55 (between stress and self-efficacy) and hence lower than typical thresholds for discriminant validity issues. We repeated the CFA with the Big 5 factors included but did not find new issues. Table 2 depicts the principal correlations between all multi-items from the second CFA.

Following Podsakoff et al. [59], we loaded all items for the measures depicted in the second CFA on a single factor. Since the resulting CFA has an unreasonably poor fit (Chi-squared = 402,182.60, df = 1,890, RMSEA = 0.10, SRMR = 0.12), a potential common method bias is unlikely. Finally, we applied Tukey HSD-post hoc tests on all measures and control variables for differences between the quartiles for respondents (first quartile = early, forth quartile = late) based on the temporal order of responses. No significant differences ($p < .05$) were found. Hence, early responses did not differ from late responses, drawing a non-response bias rather unlikely [56].

Table 2. Pearson correlation.

	Invul	Disrupt	Health	Selfeff	Info	Stress	Resp	B5Agr	BB5Cons	B5Extr	B5Open	B5Stab
**Invul**	1.00											
**Disrupt**	-0.23	1.00										
**Health**	-0.03	0.30	1.00									
**Selfeff**	0.21	-0.08	0.31	1.00								
**Info**	-0.07	0.23	0.36	0.14	1.00							
**Stress**	-0.09	0.27	-0.15	-0.55	0.01	1.00						
**Resp**	-0.15	0.32	0.41	0.09	0.37	-0.05	1.00					
**B5Agr**	-0.05	-0.03	0.26	0.37	0.11	-0.34	0.13	1.00				
**B5Cons**	-0.01	-0.01	0.34	0.41	0.16	-0.31	0.09	0.35	1.00			
**B5Extr**	0.01	0.03	0.20	0.30	0.09	-0.08	-0.02	0.51	0.18	1.00		
**B5Open**	0.07	-0.07	0.17	0.37	0.05	-0.08	0.01	0.29	0.040	0.54	1.00	
**B5Stab**	0.12	-0.05	0.22	0.55	0.10	-0.47	0.08	0.50	0.33	0.15	0.15	1.00

Notes. Invul = Invulnerability; Disrupt = Perceived disruption; Health = Health importance; Selfeff = Self-efficacy; Info = Information seeking; Stress = Perceived stress during COVID-19; Resp = Response effectiveness; B5Agr = Big 5 agreeableness; B5Cons = Big 5 conscientiousness; B5Extr = Big 5 extraversion; B5Open = Big 5 openness; B5Stab = Big 5 Stability.

https://doi.org/10.1371/journal.pone.0263022.t002

Regression analysis

Since no substantial issues with the measures or data have been found, we continued with the model approach. Linear regressions were applied for the outcome of stress with COVID-19 beliefs and self-efficacy, as well as the control variables as predictors. We opted against SEM due to the increased complexity and high number of direct and mediated effects captured by our model. Instead, for all multi-item measures, standardized factor scores from the second CFA are used. Table 3 illustrates the two models.

Investigating the outcome of perceived stress first, information seeking positively affected stress (b = 0.10, $p < 0.01$), confirming H1. Perceived disruption increased stress (b = 0.28, $p < 0.01$) and was the second-most important predictor to explain stress. Hence, H2 was confirmed. Perceived invulnerability also increased stress perception instead of weakening it (b = 0.07, $p < 0.01$), rejecting H3. In contrast, H4 was confirmed as health importance negatively affects stress as proposed (b = -0.05, $p < 0.01$). The same negative effect was found for response effectiveness (b = -0.06, $p < 0.01$). Thus, H5 was also confirmed. Finally, positive self-efficacy reduced stress perception (b = -0.57, $p < 0.01$) as the most important predictor, confirming H6. Purely explorative, younger, cisgender women, more experienced, less agreeable, more conscientious, extroverted, more open, and less stable respondents showed

Table 3. Regression results.

Outcome: Perceived stress during COVID-19				Outcome: COVID-19 related self-efficacy			
**Predictor**	***b***	***b*** 95% CI [LL, UL]	***beta***	**Predictor**	***b***	***b*** 95% CI [LL, UL]	***beta***
(Intercept)	-0.04**	[-0.06, -0.02]		(Intercept)	-0.02*	[-0.03, -0.00]	
Perceived invulnerability	0.07**	[0.06, 0.08]	0.06	Perceived invulnerability	0.15**	[0.14, 0.16]	0.14
Perceived disruption	0.28**	[0.27, 0.29]	0.28	Perceived disruption	-0.05**	[-0.06, -0.04]	-0.05
Health importance	-0.05**	[-0.06, -0.03]	-0.04	Health importance	0.06**	[0.05, 0.07]	0.06
Information seeking	0.10**	[0.09, 0.11]	0.10	Information seeking	-0.00	[-0.01, 0.01]	-0.00
Response effectiveness	-0.06**	[-0.07, -0.05]	-0.06	Response effectiveness	0.06**	[0.05, 0.07]	0.06
Self-efficacy	-0.57**	[-0.59, -0.56]	-0.56				
Age	-0.05**	[-0.06, -0.04]	-0.06	Age	0.05**	[0.04, 0.05]	0.05
Gender (cisgender women)	0.08**	[0.06, 0.10]	0.04	Gender (cisgender women)	-0.02**	[-0.04, -0.01]	-0.01
Experience (< 1 year)	-0.05*	[-0.09, -0.01]	-0.01	Experience (< 1 year)	0.04*	[0.00, 0.07]	0.01
Experience (1–5 years)	-0.04**	[-0.06, -0.01]	-0.01	Experience (1–5 years)	0.07**	[0.04, 0.09]	0.02
Experience (6–10 years)	-0.03*	[-0.07, -0.00]	-0.01	Experience (6–10 years)	0.10**	[0.07, 0.13]	0.03
Experience (> 10 years)	-0.02	[-0.05, 0.01]	-0.01	Experience (> 10 years)	0.09**	[0.07, 0.12]	0.04
Agreeableness	-0.25**	[-0.27, -0.24]	-0.22	Agreeableness	-0.17**	[-0.19, -0.16]	-0.16
Conscientiousness	0.03**	[0.02, 0.05]	0.03	Conscientiousness	0.32**	[0.31, 0.33]	0.28
Extraversion	0.12**	[0.10, 0.13]	0.12	Extraversion	0.06**	[0.05, 0.07]	0.06
Openness	0.21**	[0.19, 0.22]	0.19	Openness	0.34**	[0.33, 0.35]	0.32
Stability	-0.08**	[-0.10, -0.07]	-0.08	Stability	0.51**	[0.50, 0.52]	0.50
Model fit:				Model fit:			
R^2 = .55**				R^2 = .65**			
95% CI [.54,.55]				95% CI [.64,.65]			

Notes. A significant b-weight indicates the beta-weight is also significant. b represents unstandardized regression weights. beta indicates the standardized regression weights. LL and UL indicate the lower and upper limits of a confidence interval, respectively.

* Indicates $p < 0.05$.

** indicates $p < 0.01$.

https://doi.org/10.1371/journal.pone.0263022.t003

increased stress perception. Overall, the model explained 54.6% of the variation in perceived stress during COVID-19. Older, cisgender men, more experienced, less agreeable, more conscientious, more extraverted, more open, and more stable respondents showed increased self-efficacy.

Mediation analysis

For mediation analysis, a mediation model with the outcome self-efficacy and the COVID-19 beliefs (and control variables) as predictors was obtained. We assessed the mediation effect via bootstrapping (quasi-Bayesian percentile confidence intervals based on 1,000 replications).

Self-efficacy in turn was not driven by information seeking (b = -0.00, $p > 0.05$) and hence could not mediate its effect on stress (mediation effect = 0.00, p = 0.73, Lower 95% confidence interval [LL] = -0.00, Upper 95% confidence interval [UL] = 0.01). H7a was hence rejected. However, perceived disruption decreased self-efficacy (b = -0.05, $p < 0.01$) and was positively mediated by self-efficacy (mediation effect = 0.03, $p < 0.01$, LL = 0.02, UL = 0.03). That is, perception of disruption not only increased stress but also weakened self-efficacy, leading to increased stress. Confirming H7b, this mediation effect explains 9.2% of the absolute total effect of disruption. In contrast, self-efficacy was fostered by perceived invulnerability (b = 0.15, $p < 0.01$) and mediated the effect of invulnerability on stress (mediation effect = -0.08, $p < 0.01$, LL = -0.09, UL = -0.08). This finding indicated that direct and mediation effects were opposed to each other. While feeling invulnerable increased stress directly, it strengthened self-efficacy, which in turn reduced stress perception. Overall, the mediation effect was larger (-0.08) than the direct effect (0.07), explaining 56.2% of the absolute total effect (H7c confirmed). Health importance showed a positive effect on self-efficacy (b = 0.06, $p < 0.01$), leading to a negative mediation effect by self-efficacy on stress (mediation effect = -0.04, $p < 0.01$, LL = -0.04, UL = -0.03). Thus, direct (-0.05) and mediation effect (-0.08) jointly reduced stress perception. The latter mediation effect contributed 44.0% to the absolute total effect, confirming H7d. Finally, response effectiveness was negatively mediated by self-efficacy (mediation effect = -0.03, $p < 0.01$, LL = -0.04, UL = -0.03), due to its positive effect on self-efficacy (b = 0.06, $p < 0.01$). Again, this indicated that response effectiveness directly (-0.06) and indirectly via self-efficacy (-0.03) weakened stress perception, similar to health importance. Hence, H7e was confirmed and explained 36.0% of the absolute total effect. It should be noted that two-thirds of self-efficacy's variance was captured (64.8%). The overall effects and a comparative interpretation of direct and mediation effects are depicted in Table 4.

Table 4. Comparison of direct and mediation effects for the predictors of perceived stress.

Predictor	Direct effect on perceived stress during COVID-19	Mediation effect by self-efficacy on perceived stress during COVID-19	Comparative interpretation of direct and mediation effect
Information seeking	positive (H1✓)	not found (H7a×)	Increases stress directly
Perceived disruption	positive (H2✓)	positive (H7b✓)	Joint effects–Increases stress directly and increases stress by decreasing self-efficacy
Perceived invulnerability	positive (H3×)	negative (H7c✓)	Opposite effects–Increases stress directly but decreases stress by increasing self-efficacy
Health importance	negative (H4✓)	negative (H7d✓)	Joint effects–Decreases stress directly and decreases stress by increasing self-efficacy
Response effectiveness	negative (H5✓)	negative (H7e✓)	Joint effects–Decreases stress directly and decreases stress by increasing self-efficacy
Self-efficacy	negative (H6✓)	-	Decreases stress directly, serves as a mediator for 4 out 5 predictors

Notes. ×: Hypothesis rejected, ✓: Hypothesis confirmed.

https://doi.org/10.1371/journal.pone.0263022.t004

As an additional test, we modeled the moderation effects of the five COVID-related beliefs by self-efficacy with the outcome of perceived stress. Notwithstanding significant effects due to the sample size, two variables (perceived disruption, health importance) showed no significant moderation while three (psychological empowerment, perceived invulnerability and response effectiveness) remained significant. Since this model explained substantially less variance in stress perception (43.7% compared to 54.6), we conclude that the proposed mediation effects fitted better with the empirical data.

Discussion

The previous section depicted the results based on our initial research questions: Do the various COVID-19 related beliefs (information seeking; perceived invulnerability; perceived disruption; health importance and response effectiveness) act as predictors of perceived stress, and does the level of self-efficacy act as a mediator in reducing perceived COVID-19 related stress? From these results, five of our direct effect hypotheses were confirmed (H1, H2, H4, H5 & H6). We found that higher levels of information seeking directly led to increased perceived stress levels (H2). During the first few months since the emergence of COVID-19, little was known about it by the general public. A plethora of information was suddenly available, and an extensive amount of information was conflicting between various sources. As new evidence and data became available to medical staff, scientists and government officials, new and updated information also became available through the mainstream media. Information and updates on the global status of COVID-19 related news were reported on by most, if not all, media outlets. This made it difficult for individuals who wanted to use avoidance as a stress-coping strategy to do so. Further, an extensive amount of this information was negative, inducing a sense of fear and anxiety among most individuals. Since self-efficacy was not driven by information seeking, the increased level of information likely confounded individuals, inducing a feeling of not being in control and less efficient to deal with COVID-19 related issues.

We further found that higher perceived levels of disruption are associated with higher perceived stress during COVID-19 (H2). These results are in line with studies by Jones and Butler [31], Picou et al. [32], and Elliott [33]. Perceived disruption acted as the 2nd most important predictor to explain stress. Generally, individuals who consider contracting COVID-19 as disruptive to their social and everyday health would experience higher levels of perceived stress. Disruption causes inconvenience and upsets the lives of individuals. Furthermore, disruptive situations prohibit individuals from continuing with life as 'usual' or normal, leading to a feeling of not being in control. Likewise, this state of feeling out of control creates a heightened sense of stress. This result aligns with previous findings on disruption and stress and supports the notion that individuals like to be in control, especially in unstable situations [60, 61]. Considering the indirect effect, perceived disruption caused by COVID-19 had a negative effect on self-efficacy, which increased perceived stress. Thus, the fear of disruption impacts stress directly and reduces self-efficacy, which leads to additional stress levels.

We further suggested that higher health importance would be associated with lower stress (H4). Our assumption was confirmed. This finding is in line with the WHO [43], which states that individuals with high health importance may be less stressed as they may be more aware of the consequences of COVID-19, thus acting more responsible or precautious. Hence, health importance does not amplify stress perception; it helps to mitigate it directly or by increasing self-efficacy. This implies that people who consider their health important, take action to prevent contracting COVID-19 and actively take care of their health will be less stressed and have a higher level of self-efficacy, leading to further reduction in stress levels.

Our findings further confirmed that higher response effectiveness leads to lower levels of stress (H5). Thus, simply put, individuals who believe that protocols such as the washing of hands, wearing a mask, avoiding crowds and social distancing could reduce the spread of COVID-19 were naturally less stressed. This finding is similar to that of Vagni [11] and Oh et al. [44], who confirmed that the prevalence of COVID-19 protocols reduced stress. Alike health importance, perceiving responses as more effective helps to reduce stress directly and fosters self-efficacy.

Not surprising, the variable that had the highest predicting power over our outcome variable–stress–was self-efficacy (H6). Confirming research such as Zajacova et al. [10], Shahrour and Dardas [10] and Mo et al. [9] state that higher self-efficacy levels lead to reduced stress levels. Karademas and Kalantzi-Azizi [49] found that self-efficacy influenced how individuals perceived threats and challenges, thus providing a sense of control and reducing stress. Moreover, our findings confirm the key role of self-efficacy for the appraisal of challenging conditions such as COVID-19 [49]. Our findings correspond to those of Chemers et al. [51], who state that self-efficacy is influenced by certain predictors, converting it into the role of a mediator. This emphasizes the importance of self-efficacy in those individuals who are more confident and able to exert control over situations may be less stressed.

The hypothesis relating to invulnerability was rejected (H3). Based on existing literature, we assumed that higher perceived invulnerability would be associated with lower perceived stress. However, we found a positive relationship. This can refer to aspects of "COVID-19 denial" [62]. Individuals may disclose that they are less vulnerable to COVID-19 but still accept that stressing factors apply. In line with that, stress can be understood as a heightened state of emotions resulting from socio-environmental demands; it becomes plausible that social and economic consequences also stress the ones believing to be invulnerable. In essence, a pandemic not only causing health issues, but even the most durable also are upset and stressed by non-health-related consequences of COVID-19. However, and this is in line with our assumptions (H7c), believing to be invulnerable still increases self-efficacy, causing the opposing direct and mediated effects.

Conclusion

As stated, existing literature suggests that certain environmental stressors may lead to increased stress [21]. The COVID-19 pandemic led to numerous stressors, and from the start of the pandemic, individuals were faced with increased stress levels due to several health, social and economic fears. This emphasizes the importance of continued research on the topic of COVID-19 related psychological factors such as stress and how to implement effective coping strategies given the current situation. The literature further indicates that self-efficacy can positively increase a person's ability to cope with stress [46]. For instance, stress-coping methods such as a sense of coherence, locus of control, self-belief, and self-efficacy could reduce stress [63]. Following Bandura [64], this research confirms that self-efficacy is one of the most important factors to manage stress levels.

Consequently, our study found that various COVID-19 related beliefs (information seeking; perceived invulnerability; perceived disruption; health importance and response effectiveness) distinctly act as predictors of perceived stress. Moreover, self-efficacy mediates these distinct effects on stress perception. We, therefore, conclude that focusing on certain COVID-19 beliefs, for example, focusing on health importance, protection protocols and feeling less vulnerable as a result of this, may improve self-efficacy, which may be valuable to manage stress in turbulent situations like we are currently in the midst of. While most predictors jointly shape stress levels directly and via the mediator of self-efficacy, perceived

invulnerability seems to be a double-edged sword. On the one hand, feeling invulnerable increases self-efficacy which then return anxiety and stress. On the other hand, feeling invulnerable still induces stress, presumably because non-health related issues like social (e.g., social distancing, loneliness) and economic (e.g., job security, income) aspects cannot be denied. To the best of our knowledge, this study is the first to address this differential effect.

Study limitations

Although our findings contribute to the emerging literature on COVID-19 psychological related research, it is not without limitations. Firstly, since our study sample included several nationalities, some cultural differences may be present. Given our focus on a generalizable large and broad sample to investigate the principal effects of beliefs, self-efficacy and stress, we omitted detailed analyses as well as validation checks for sub-samples and call for future research to isolate, e.g., cultural (e.g., long-term orientation), economic (e.g., GDP) and demographic differences (e.g., age and gender). Second, variations of time are not considered for the same reasons as explained before. Particularly, all measures may be different given the various waves of infections. Since the epidemic waves occurred temporally different in various countries and even show local differences that might be present in our sample, we were unable to track this issue, implying a venture for subsequent research. Third, the present research focused on a limited set of variables. Other mediators (e.g., attitudes towards the pandemic, vaccination), as well as downstream intentions and behaviors (e.g., restarting a business, resuming a job, vaccination intent), can furtherly complete the research puzzle. Finally, we acknowledge that some additional limitations may be present such as the use of Computer Assisted Web Interviewing (CAWI) data collection and non-probability sampling.

Supporting information

S1 Appendix. Scale items [65–67].
(DOCX)

Author Contributions

Conceptualization: Natanya Meyer, Thomas Niemand.

Data curation: Thomas Niemand, Andrés Davila.

Formal analysis: Thomas Niemand.

Investigation: Natanya Meyer, Thomas Niemand.

Methodology: Natanya Meyer, Thomas Niemand.

Project administration: Natanya Meyer, Andrés Davila, Sascha Kraus.

Supervision: Sascha Kraus.

Validation: Thomas Niemand.

Visualization: Andrés Davila, Sascha Kraus.

Writing – original draft: Natanya Meyer, Thomas Niemand.

Writing – review & editing: Natanya Meyer, Sascha Kraus.

References

1. WHO. Coronavirus disease (COVID-19)–World Health Organization. 2020 [cited 17 Aug 2021]. Available: https://www.who.int/emergencies/diseases/novel-coronavirus-2019
2. Worldometer. COVID Live Update. 2021 [cited 17 Aug 2021]. Available: https://www.worldometers.info/coronavirus/
3. Cullen W, Gulati G, Kelly BD. Mental health in the COVID-19 pandemic. QJM: An International Journal of Medicine. 2020; 113: 311–312. https://doi.org/10.1093/qjmed/hcaa110 PMID: 32227218
4. Murthy RS, Lakshminarayana R. Mental health consequences of war: a brief review of research findings. World Psychiatry. 2006; 5: 25–30. Available: https://www.ncbi.nlm.nih.gov/pmc/articles/PMC1472271/ PMID: 16757987
5. Pfefferbaum B, North CS. Mental Health and the Covid-19 Pandemic. New England Journal of Medicine. 2020; 383: 510–512. https://doi.org/10.1056/NEJMp2008017 PMID: 32283003
6. Taylor S. The Psychology of Pandemics: Preparing for the Next Global Outbreak of Infectious Disease. Cambridge Scholars Publishing; 2019.
7. Clark C, Davila A, Regis M, Kraus S. Predictors of COVID-19 voluntary compliance behaviors: An international investigation. Global Transitions. 2020; 2: 76–82. https://doi.org/10.1016/j.glt.2020.06.003 PMID: 32835202
8. Bandura A. Self-efficacy: Toward a unifying theory of behavioral change. Psychology. 1977; 84: 191–215. Available: https://doi.apa.org/doiLanding?doi=10.1037%2F0033-295X.84.2.191
9. Mo Y, Deng L, Zhang L, Lang Q, Pang H, Liao C, et al. Anxiety of Nurses to support Wuhan in fighting against COVID-19 Epidemic and its Correlation With Work Stress and Self-efficacy. Journal of Clinical Nursing. 2021; 30: 397–405. https://doi.org/10.1111/jocn.15549 PMID: 33141987
10. Shahrour G, Dardas LA. Acute stress disorder, coping self-efficacy and subsequent psychological distress among nurses amid COVID-19. Journal of Nursing Management. 2020; 28: 1686–1695. https://doi.org/10.1111/jonm.13124 PMID: 32767827
11. Vagni M, Maiorano T, Giostra V, Pajardi D. Coping With COVID-19: Emergency Stress, Secondary Trauma and Self-Efficacy in Healthcare and Emergency Workers in Italy. Frontiers in Psychology. 2020; 11: 2294. https://doi.org/10.3389/fpsyg.2020.566912 PMID: 33013603
12. Torrès O, Fisch C, Mukerjee J, Lasch F, Thurik R. Health Perception of French SME Owners During the 2020 COVID-19 Pandemic. 2020; 18.
13. Lathabhavan R, Barami A N, Kurikkal MPMM, Manoj N. Mental health concerns of small business entrepreneurs in India due to COVID-19 financial distress. Asian J Psychiatr. 2021; 64: 102774. https://doi.org/10.1016/j.ajp.2021.102774 PMID: 34274815
14. Lazarus R. Psychological Stress and the Coping Process. New York: McGraw-Hill; 1966.
15. Pearlin L. Role strains and personal stress. Psychosocial Stress: Trends in Theory and Research. New York: Academic Press; 1983. pp. 3–32.
16. Menaghan E. Individual coping efforts: Moderators of the relationship between life stress and mental health outcomes. Psychosocial Stress: Trends in Theory and Research. New York: Academic Press; 1983. pp. 157–191.
17. Gmelch W. Coping with facukty stress. California: Sage Publications Inc.; 1993.
18. Selye H. The stress of life. New York: McGraw-Hill; 1956.
19. CDC. Coping with Stress. 2021 [cited 17 Aug 2021]. Available: https://www.cdc.gov/mentalhealth/stress-coping/cope-with-stress/index.html
20. Holmes TH, Rahe RH. The social readjustment rating scale. Journal of Psychosomatic Research. 1967; 11: 213–218. https://doi.org/10.1016/0022-3999(67)90010-4 PMID: 6059863
21. Aneshensel CS. Social Stress: Theory and Research. Annu Rev Sociol. 1992; 18: 15–38. https://doi.org/10.1146/annurev.so.18.080192.000311
22. Chen P, Spector P. Negative affectivity as the underlying cause of correlations between stressors and strains. Journal of Applied Psychology. 1991; 76: 398–407. Available: https://doi.apa.org/doiLanding?doi=10.1037%2F0021-9010.76.3.398 PMID: 1864813
23. Johnson J. Cancer-related information seeking. NY: Hampton Press; 1997.
24. Case DO. Looking for information: a survey of research on information seeking, needs, and behavior. 2nd ed. Amsterdam; Boston: Elsevier/Academic Press; 2007.
25. Brashers DE, Goldsmith DJ, Hsieh E. Information Seeking and Avoiding in Health Contexts. Human Comm Res. 2002; 28: 258–271. https://doi.org/10.1111/j.1468-2958.2002.tb00807.x

26. Yang ZJ, Kahlor L. What, Me Worry? The Role of Affect in Information Seeking and Avoidance. Science Communication. 2013; 35: 189–212. https://doi.org/10.1177/1075547012441873

27. Moreno A, Fuentes-Lara C, Navarro C. Covid-19 communication management in Spain: Exploring the effect of information-seeking behavior and message reception in public's evaluation. EPI. 2020; 29. https://doi.org/10.3145/epi.2020.jul.02

28. Lambert SD, Loiselle CG. Health Information—Seeking Behavior. Qual Health Res. 2007; 17: 1006–1019. https://doi.org/10.1177/1049732307305199 PMID: 17928475

29. Brashers DE. Communication and Uncertainty Management. Journal of Communication. 2001; 51: 477–497. https://doi.org/10.1111/j.1460-2466.2001.tb02892.x

30. Cambridge Dictionary. Meaning of disruption. Cambridge Dictionary. United Kingdom: Cambridge University Press; 2021. Available: https://dictionary.cambridge.org/dictionary/english/disruption

31. Jones AP, Butler MC. A Role Transition Approach to the Stresses of Organizationally Induced Family Role Disruption. Journal of Marriage and Family. 1980; 42: 367–376. https://doi.org/10.2307/351234

32. Picou JS, Gill DA, Dyer CL, Curry EW. Disruption and stress in an Alaskan fishing community: initial and continuing impacts of the Exxon Valdez oil spill. Industrial Crisis Quarterly. 1992; 6: 235–257. https://doi.org/10.1177/108602669200600305

33. Elliott G. Generation COVID and the Impact of Lockdown. Research Matters. 2021 [cited 17 Aug 2021]. Available: https://eric.ed.gov/?id=EJ1294096

34. Carney A, Graf AS, Hudson G, Wilson E. Age Moderates Perceived COVID-19 Disruption on Well-Being. The Gerontologist. 2021; 61: 30–35. https://doi.org/10.1093/geront/gnaa106 PMID: 32808660

35. Adger WN. Vulnerability. Global Environmental Change. 2006; 16: 268–281. https://doi.org/10.1016/j.gloenvcha.2006.02.006

36. Millstein SG, Halpern–Felsher BL. Judgments about Risk and Perceived Invulnerability in Adolescents and Young Adults. Journal of Research on Adolescence. 2002; 12: 399–422. https://doi.org/10.1111/1532-7795.00039

37. Ferrer RA, Klein WM. Risk perceptions and health behavior. Current Opinion in Psychology. 2015; 5: 85–89. https://doi.org/10.1016/j.copsyc.2015.03.012 PMID: 26258160

38. Wise T, Zbozinek TD, Michelini G, Hagan CC, Mobbs D. Changes in risk perception and self-reported protective behaviour during the first week of the COVID-19 pandemic in the United States. Royal Society Open Science. 7: 200742. https://doi.org/10.1098/rsos.200742 PMID: 33047037

39. Harper CA, Satchell LP, Fido D, Latzman RD. Functional Fear Predicts Public Health Compliance in the COVID-19 Pandemic. Int J Ment Health Addiction. 2020 [cited 18 Aug 2021]. https://doi.org/10.1007/s11469-020-00281-5 PMID: 32346359

40. Kuang J, Ashraf S, Das U, Bicchieri C. Awareness, Risk Perception, and Stress during the COVID-19 Pandemic in Communities of Tamil Nadu, India. International Journal of Environmental Research and Public Health. 2020; 17: 1–11. https://doi.org/10.3390/ijerph17197177 PMID: 33007992

41. Livingston IL. Stress and health dysfunctions: The importance of health education. Stress Medicine. 1988; 4: 155–161. https://doi.org/10.1002/smi.2460040308

42. Keller A, Litzelman K, Wisk L, Maddox T, Cheng E, Creswell P, et al. Does the perception that stress affects health matter? The association with health and mortality. Health Psychology. 2012; 31: 677–684. https://doi.org/10.1037/a0026743 PMID: 22201278

43. WHO. Health inequity and the effects of COVID-19 Assessing, responding to and mitigating the socioeconomic impact on health to build a better future. 2020. Report No.: WHO/EURO:2020-1744-41495-56594. Available: https://apps.who.int/iris/bitstream/handle/10665/338199/WHO-EURO-2020-1744-41495-56594-eng.pdf

44. Oh N, Hong N, Ryu DH, Bae SG, Kam S, Kim K-Y. Exploring Nursing Intention, Stress, and Professionalism in Response to Infectious Disease Emergencies: The Experience of Local Public Hospital Nurses During the 2015 MERS Outbreak in South Korea. Asian Nursing Research. 2017; 11: 230–236. https://doi.org/10.1016/j.anr.2017.08.005 PMID: 28991605

45. Walton M, Murray E, Christian MD. Mental health care for medical staff and affiliated healthcare workers during the COVID-19 pandemic. Eur Heart J Acute Cardiovasc Care. 2020; 9: 241–247. https://doi.org/10.1177/2048872620922795 PMID: 32342698

46. Cai H, Tu B, Ma J, Chen L, Fu L, Jiang Y, et al. Psychological Impact and Coping Strategies of Frontline Medical Staff in Hunan Between January and March 2020 During the Outbreak of Coronavirus Disease 2019 (COVID-19) in Hubei, China. Med Sci Monit. 2020; 26: 1–16. https://doi.org/10.12659/MSM.924171 PMID: 32291383

47. Zajacova A, Lynch SM, Espenshade TJ. Self-Efficacy, Stress, and Academic Success in College. Res High Educ. 2005; 46: 677–706. https://doi.org/10.1007/s11162-004-4139-z

48. Netz Y, Wu M, Becker B, Tenenbaum G. Physical Activity and Psychological Well-Being in Advanced Age: A Meta-Analysis of Intervention Studies. Psychology in Aging. 2005; 20: 272–284. Available: https://doi.apa.org/doiLanding?doi=10.1037%2F0882-7974.20.2.272 PMID: 16029091

49. Karademas EC, Kalantzi-Azizi A. The stress process, self-efficacy expectations, and psychological health. Personality and Individual Differences. 2004; 37: 1033–1043. https://doi.org/10.1016/j.paid.2003.11.012

50. Lazarus R, Folkman S. Stress, Appraisal, and Coping. New York: Springer; 1984.

51. Chemers M, Hu L, Garcia B. Academic self-efficacy and first year college student performance and adjustment. Journal of Educational Psychology. 2001; 93: 55–64. https://doi.apa.org/doiLanding?doi=10.1037%2F0022-0663.93.1.55

52. Meyer N, Niemand T, Davila A, Kraus S. Biting the bullet: When self-efficacy mediates the stressful effects of COVID-19 beliefs. Mendeley Data. 2021;1. https://doi.org/10.17632/b8zxmw5z3g.1

53. Cohen S, Kamarck T, Mermelstein R. Perceived stress scale. Measuring stress: A guide for health and social scientists. Menlo Park, USA: Mind Garden, Inc.; 1994.

54. Prigge J-K, Dietz B, Homburg C, Hoyer WD, Burton JL. Patient empowerment: A cross-disease exploration of antecedents and consequences. International Journal of Research in Marketing. 2015; 32: 375–386. https://doi.org/10.1016/j.ijresmar.2015.05.009

55. John OP, Srivastava S. The Big Five Trait taxonomy: History, measurement, and theoretical perspectives. Handbook of personality: Theory and research, 2nd ed. New York, NY, US: Guilford Press; 1999. pp. 102–138.

56. Armstrong JS, Overton TS. Estimating nonresponse bias in mail surveys. Journal of Marketing Research. 1977; 14: 396–402.

57. Niemand T, Mai R. Flexible cutoff values for fit indices in the evaluation of structural equation models. Journal of the Academy of Marketing Science. 2018; 46: 1148–1172.

58. Rönkkö M, Cho E. An Updated Guideline for Assessing Discriminant Validity. Organizational Research Methods. 2020; 1094428120968614.

59. Podsakoff PM, MacKenzie SB, Lee J-Y, Podsakoff NP. Common method biases in behavioral research: a critical review of the literature and recommended remedies. Journal of Applied Psychology. 2003; 88: 879–903. https://doi.org/10.1037/0021-9010.88.5.879 PMID: 14516251

60. Spreitzer GM. Psychological Empowerment in the Workplace: Dimensions, Measurement, and Validation. The Academy of Management Journal. 1995; 38: 1442–1465. https://doi.org/10.2307/256865

61. Spreitzer G, Quinn R. A company of leaders: Five disciplines for unleashing the power in your workforce. San Francisco, California, USA: Jossey-Bass; 2001.

62. Miller BL. Science denial and COVID conspiracy theories: potential neurological mechanisms and possible responses. JAMA. 2020; 324: 2255–2256. https://doi.org/10.1001/jama.2020.21332 PMID: 33136157

63. Holahan C, Moos R, Schaefer J. Coping, stress resistance, and growth: Conceptualizing adaptive functioning. 1st ed. Handbook of coping: Theory, research, applications. 1st ed. New York: Wiley; 1996.

64. Bandura A. Social foundations of thought and action: A social cognitive theory. Englewood Cliffs: PrenticeHall; 1986.

65. Rhodes S, Arceo R. Developing and testing measures predictive of hepatitis A vaccination in a sample of men who have sex with men | | Oxford Academic. Health Education Research. 2004; 19: 272–283. Available: https://academic.oup.com/her/article/19/3/272/642236 https://doi.org/10.1093/her/cyg026 PMID: 15140847

66. Gerend MA, Shepherd JE, Monday KA. Behavioral Frequency Moderates the Effects of Message Framing on HPV Vaccine Acceptability. Annals of Behavioral Medicine. 2008; 35: 221–229. https://doi.org/10.1007/s12160-008-9024-0 PMID: 18347893

67. Van Empelen P, Schaalma H, Kok G, Jansen M. Predicting condom use with casual and steady sex partners among drug users. Health Education Research. 2001; 16: 293–305. Available: https://academic.oup.com/her/article/16/3/293/703097 https://doi.org/10.1093/her/16.3.293 PMID: 11497113
